# Preanalytical conditions can interfere with *M. tuberculosis* detection by PCR in respiratory samples

**DOI:** 10.6061/clinics/2017/e410

**Published:** 2018-11-16

**Authors:** Gabriela Gaspar Carnevale, Francisco Suso Vargas, Hélio Hehl Caiaffa-Filho, Milena Marques Pagliarelli Acencio, Lia Junqueira Marçal, Roberta Karla Barbosa Sales, Lisete Ribeiro Teixeira, Leila Antonangelo

**Affiliations:** I Divisao Pulmonar, Instituto do Coracao (InCor), Hospital das Clinicas HCFMUSP, Faculdade de Medicina, Universidade de Sao Paulo, Sao Paulo, SP, BR; II Laboratorio de Investigacao Medica (LIM 03), Hospital das Clinicas HCFMUSP, Faculdade de Medicina, Universidade de Sao Paulo, Sao Paulo, SP, BR

**Keywords:** Tuberculosis, Sputum, Bronchoalveolar Lavage, Pleural Fluid, Polymerase Chain Reaction

## Abstract

**OBJECTIVES:**

Tuberculosis is one of the most prevalent infections in humans. Although culture is the reference for diagnosis, its sensitivity is compromised, especially in paucibacillary samples. Because polymerase chain reaction (PCR) amplifies mycobacterial DNA, it is more sensitive than culture for the diagnosis of *Mycobacterium tuberculosis* (Mtb). However, its performance can be affected by intrinsic sample inhibitors and by the extraction/detection techniques used.

**METHODS:**

We evaluated the influence of preanalytical conditions on Mtb detection in samples of sputum (SPU), bronchoalveolar lavage (BAL), and pleural fluid (PF) using combinations of extraction/detection methods. Respiratory samples were prepared to contain different concentrations of red blood cells and nucleated cells to which increasing amounts of Mtb colonies were inoculated and submitted to PCR.

**RESULTS:**

Up to 10^2^ CFU/ml of Mtb were detected in the SPU in all methods, except for the Roche extraction/detection method, regardless of the preanalytical sample condition. In BAL samples, medium and high concentrations of cells and high concentrations of red blood cells contributed to a lower Mtb detection, regardless of the extraction method used. In PF, red blood cells were the variable that most interfered with Mtb detection, with better recovery (10^2^ CFU/ml) observed with the Qiagen/Nanogen combination.

**CONCLUSION:**

The choice of Mtb extraction and detection method is of fundamental importance for PCR analytical sensitivity, especially when paucibacillary samples and/or samples containing potential PCR inhibitors are analyzed.

## INTRODUCTION

Tuberculosis (TB) is one of the most prevalent infections in humans. It is estimated that one-third of the world's population is infected with the bacillus. Brazil ranks 16^th^ among the 22 countries responsible for 80% of all tuberculosis cases in the world 1.

Ziehl Neelsen's smear microscopy is the first approach used in clinical laboratories for the diagnosis of clinical TB. However, its limited sensitivity and specificity make it less reliable than other methods, especially in cases of poor sputum (SPU) quality and low mycobacterial content. Culture is still the gold standard for TB diagnosis because it is more sensitive than microscopy and is highly specific 2, but the method is not sufficiently rapid.

In pleural TB, the confirmatory diagnosis is also difficult since the scarce number of bacilli provides a positive bacilloscopy and culture lower than 5% and 30%, respectively 3-6.

Polymerase chain reaction (PCR) has the potential to improve the diagnosis of TB, although it also has diagnostic limitations. The high false-negative rate (approximately 20%) is generally associated with the presence of intrinsic and extrinsic inhibitors of Taq polymerase 8. However, false-positive results are frequently due to sample contamination with mycobacterial DNA or PCR amplicons 7.

According to the literature, PCR tests performed on SPU, bronchoalveolar lavage (BAL), or pleural fluid (PF) samples for TB diagnosis exhibit variable sensitivity (11 to 81%) depending on the number of bacilli in the samples, the type of biological matrix, the presence of intrinsic and extrinsic sample inhibitors and the detection methods used 8-13. The target sequence selected and the method of DNA extraction used are also important in PCR performance 12.

Considering the reported factors that affect Mtb amplification, the present study was designed to evaluate whether intrinsic conditions of the sample, such as hemorrhage and cell content, can influence Mtb detection in respiratory specimens.

## MATERIAL AND METHODS

This study was undertaken at the Pleura Laboratory and at the Cytology and Molecular Biology Laboratories of the Hospital das Clinicas da Faculdade de Medicina da Universidade de Sao Paulo (HCFMUSP) after approval by the Ethics Committee (0587/11) and by the FAPESP under number 11/50842-2.

After informed consent was obtained, samples of SPU, BAL and PF from patients not infected by Mtb were collected through induced expectoration, bronchoscopy or thoracentesis, respectively. All patients had clinical indications for the procedures and lacked any clinical history or physical examination results that suggested a TB diagnosis.

The samples were sent for cell and erythrocyte counting, smear microscopy and culture to confirm the absence of TB infection. To test possible preanalytical interference in PF, one sample was enough for the preparation of test tubes, in light of the large volume of fluid obtained in thoracentesis. To prepare the BAL and SPU test tubes, sample pools were obtained from 48 patients and 82 patients, respectively. The samples were mixed and homogenized in sufficient volume and were stored until PCR analysis.

To determine the Mtb detection threshold, samples were prepared *in vitro* to contain different concentrations of interference, simulating the preanalytical condition of respiratory samples frequently received in the laboratory routine. An increasing number of colony-forming units (CFU) of mycobacteria was added to these prepared sample tubes.

### Sample preparation according to the preanalytical variables

As hemorrhage and cell content can influence Mtb detection in respiratory specimens, samples were prepared in groups according to the concentration of nucleated cells and erythrocytes, potential preanalytical variables of interference in PCR. Cell and red blood cell concentrations were adjusted with white blood cells and/or red blood cells from the peripheral blood of patients.

A mixed group was also prepared in which the samples were adjusted to contain the maximum concentrations of these variables (Table 1).

Samples were tested in triplicate. The analytical sensitivity of detection took into account the lowest value detected simultaneously in all of the triplicate samples. If detection failed in any of the triplicate tubes, the previous value was considered. This approach was used to guarantee the limit of test detection.

### Preparation of the serial concentrations of *M. tuberculosis*

*Mycobacterium tuberculosis* strains isolated at the Microbiology Laboratory from a patient with TB were used in the assay. The strains were mixed in sterile water until 0.5 on the McFarland scale was reached (1.5 x 10^8^ CFU/ml). From this solution, serial dilutions of the microbiological agent (from 1.5 x 10^6^ to 1.5 x 10^1^ CFU/ml) were inoculated into test tubes. A tube not inoculated with bacteria was used as a negative control. The samples were heated to a temperature of 100°C for 10 minutes to ensure that the agent would lose its virulence.

Finally, we tested the recovery efficiency of *M. tuberculosis* in the pleural exudate of a patient with a clinical and histological diagnosis of pleural tuberculosis but with a negative smear and culture in the PF. In aliquots of this sample, serial dilutions of mycobacteria (1.5 x 10^6^ at 1.5 x 10^1^ CFU/ml) in triplicate were prepared for each dilution, and the samples were subjected to PCR using the extraction/detection techniques described below.

### Analytical methods

For PCR, DNA was extracted using the QIAamp® DNA Mini Kit (Qiagen, Hilden, Germany) using detergents and silica columns and the AMPLICOR® Respiratory Specimen Preparation Kit (Roche Molecular Systems, Inc., Branchburg, NJ, USA) using only detergents. The input volumes (1 ml) were the same for all reactions.

The DNA extracted was amplified and detected by three methods:

1. Cobas® TaqMan® MTB Test (Roche Molecular Systems, Inc., Branchburg, NJ, USA) – this is a real-time PCR method validated for liquefied, decontaminated and concentrated human respiratory samples, including SPU and BAL. The Cobas® TaqMan® MTB Test uses *Mycobacterium* specific primers to define a sequence for the gene 16S rRNA. The detection limit of the test is 0.46 CFU/PCR or approximately 18 CFU/ml, according to the manufacturer;
2. MTB Q – PCR Alert Kit (Nanogen Advanced Diagnosis, Trezzano, Italy) – this is also a real-time PCR method validated for SPU, urine and exudates. In this approach, the IS6110 genomic region of Mtb is amplified. This assay has a detection limit of 10 genomes of *M. tuberculosis* per reaction;
3. “In-house” method using 100 µM of specific primers to define a sequence for the gene 16S rRNA. The detection limit of this test is approximately 100 CFU/ml.

The following combinations were tested: Roche extraction/Roche detection (R/R); Roche extraction/Nanogen detection (R/N); Roche extraction/“in-house” detection (R/IH); Qiagen extraction/Roche detection (Q/R); Qiagen extraction/Nanogen detection (Q/N); and Qiagen extraction/“in-house” detection (Q/IH). The choice for these combinations was based on our routine practice and the kind of samples tested in this study.

## RESULTS

### Sputum

**Figure 1:**
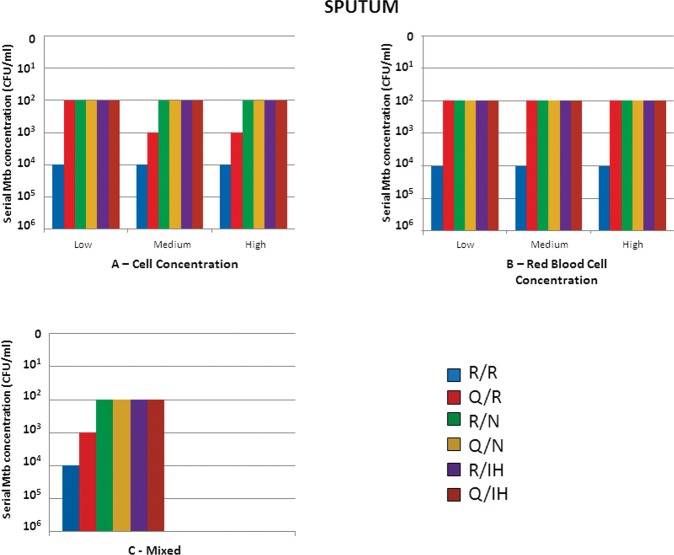
Results of Mtb detection in sputum samples according to the concentration of preanalytical variables and different combinations of extraction/detection methods

Regardless of cell concentration, up to 10^2^ CFU/ml was detected using the R/N, Q/N, R/IH and Q/IH combinations. The R/R combination showed the worst performance, detecting only up to 10^4^ CFU/ml. Similar performance was observed in the erythrocytes and mixed groups (Figures 1 A, B and C).

### Bronchoalveolar lavage

**Figure 2:**
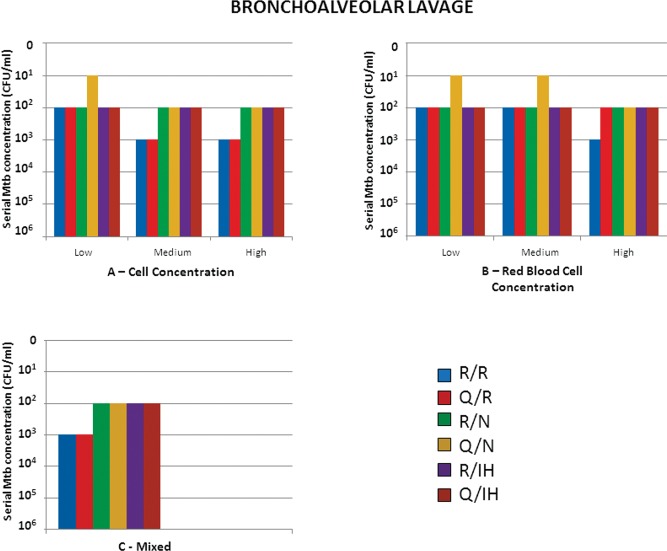
Results of Mtb detection in bronchoalveolar samples according to the concentration of preanalytical variables and different combinations of extraction/detection methods

For BAL, we observed that up to 10^2^ CFU/ml could be detected in samples with low cellularity, regardless of the extraction/detection technique used, although the Q/N combination was able to detect up to 10^1^ CFU/ml. However, in cases of medium and high cell concentrations, only 10^3^ CFU/ml was detected with the Roche assay (Figure 2A).

When evaluating the variable hemorrhage, the best detection was obtained with the combination Q/N (10^1^ CFU/ml). In samples containing the highest red blood cell concentration, the least satisfactory performance was obtained with the R/R combination, which detected up to 10^3^ CFU/ml (Figure 2B). In the mixed BAL group (high cell and erythrocyte concentrations), the detection of up to 10^2^ CFU/ml was obtained with all combinations with the exception of R/R (Figure 2C).

### Pleural fluid

**Figure 3:**
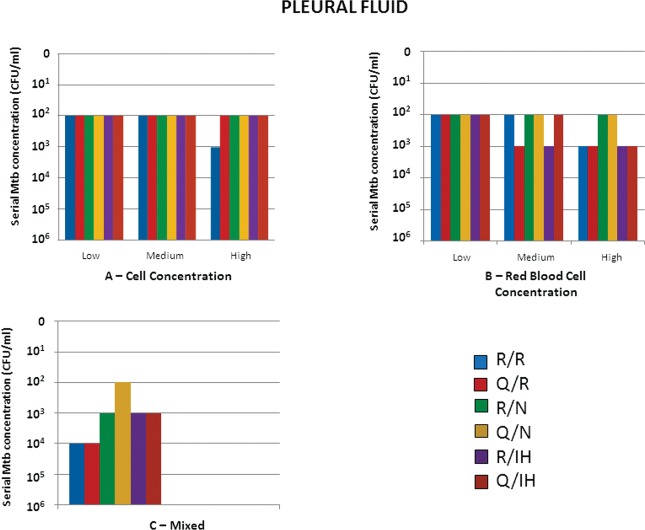
Results of Mtb detection in pleural samples according to the concentration of preanalytical variables and different combinations of extraction/detection methods

Cellularity had a small influence on Mtb detection in PF samples. Up to 10^2^ CFU/ml was detected with most of the extraction/detection methods used, except for the R/R combination, which detected up to 10^3^ CFU/ml in samples with high cellularity (Figure 3A)

In PF samples, the concentration of erythrocytes was the most likely factor interfering with Mtb detection. Considering the medium PF erythrocyte concentration, Q/R and R/IH combinations detected up to 10^3^ CFU/ml. In samples containing more than 50,000 red blood cells/ml, the detection of up to 10^2^ CFU/ml was possible only with the Nanogen assay (Figure 3B).

In samples from the mixed group, which have similar characteristics to tuberculous exudate, only the Q/N combination was capable of detecting up to 10^2^ CFU/ml (Figure 3C).

**Figure 4:**
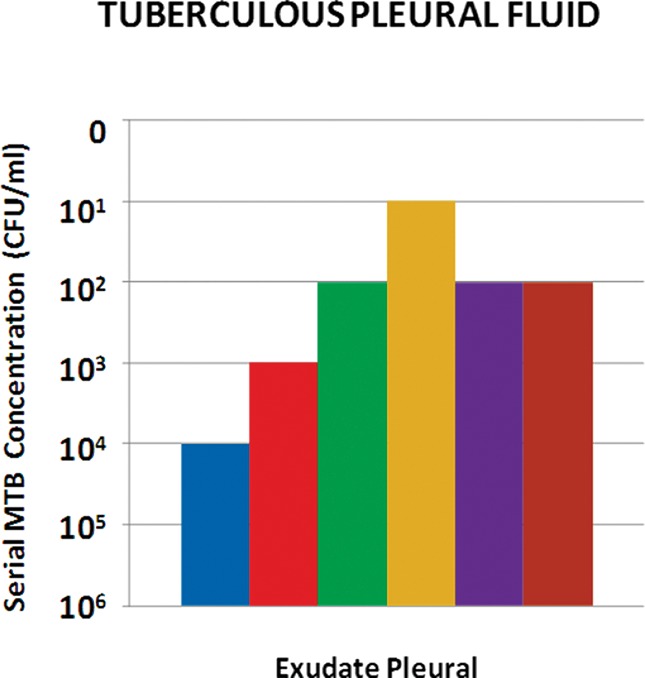
Results of Mtb detection in pleural fluid from a case of pleural tuberculosis with different combinations of extraction/detection methods

The sample from the case of pleural tuberculosis showed the following laboratory profile: 860 cells/mm^3^ with 94% lymphocytes; 480 red blood cells/mm^3^; 5.1 g/dl protein; and an adenosine deaminase (ADA) level of 67.6 U/l. PF smear microscopy and culture for Mtb were negative, and the parietal pleura biopsy showed chronic granulomatous inflammation with caseous necrotic foci, AFB negative. In this sample, the combination Q/R detected 10^2^ CFU/ml in only one of the triplicate tubes, while with the Q/N combination, the detection of 10^1^ CFU/ml was possible in two of the triplicate tubes (Figure 4).

## DISCUSSION

In this study, we examined whether preanalytical conditions of respiratory samples may interfere with the performance of molecular tests. SPU, BAL and PF samples were prepared to contain increasing concentrations of PCR interference (erythrocytes and cells). In these tubes, known concentrations of mycobacteria were inoculated, mimicking Mtb-positive clinical specimens.

We observed that in SPU samples, the number of cells and red blood cells rarely interfered with the detection of *M. tuberculosis*, with the exception of medium and high cell concentrations with the Q/R combination. In BAL samples, a high concentration of cells and erythrocytes interfered with mycobacterial detection in most of the combinations used. For PF samples, the concentration of red blood cells was the variable that most interfered with the detection, except in combinations that used the Nanogen kit for detection. Cell concentration had little effect on Mtb detection.

Tests based on PCR allow rapid and reliable detection of *M. tuberculosis* DNA, which is essential for the early diagnosis and clinical management of patients 15. The use of real-time PCR is recommended because in addition to being more rapid, sensitive and specific, it has a low rate of sample contamination 16.

However, the performance of PCR assays can be affected by other factors, including the difficult amplification of mycobacterial gene content due to the complexity of its lipid wall 17,18, the low number of microorganisms in the sample, the presence of intrinsic or extrinsic PCR inhibitors^16^, and the types of primers used in the reaction 7,14.

To simulate most amplification problems, Restrepo B.I. et al. 7 used blood as a model for the PCR assay, since blood is paucibacillary, has internal Taq polymerase inhibitors, and has an excess of genomic DNA. The authors reported variable sensitivity (33-95%), depending on the method of extraction and the type of PCR assay used.

However, despite the promising results of PCR for diagnostic use, choosing the best extraction method to efficiently detect mycobacterial DNA in paucibacillary samples remains a challenge 7,18,19_._

The sensitivity of a PCR assay is also affected by the complex environment of the biological matrix in which the mycobacteria are present 7. Suffys P. et al. 20, when considering the low PCR sensitivity in samples of SPU with positive smear (67%), suggested that mycobacterial DNA degradation or inhibition had occurred during sample processing. The authors tested 36 SPU samples in which they mixed pure *M. tuberculosis* DNA at a final concentration of 10 ng/ml and performed PCR. The inhibition was evaluated by comparing the intensity of the amplified product with three PCRs without the addition of DNA. The authors concluded that PCR was inhibited in a significant number of samples, highlighting the importance of the extraction method used in eliminating possible sample interference.

In accordance with the literature, we analyzed the performance of two extraction methods, considering that the ideal extraction should minimize the influence of sample interference. In general, for the 3 types of samples tested, the best detection results were obtained when using the Qiagen extraction method, regardless of the preanalytical sample conditions. As this method is based on silica columns, it may present an advantage for better DNA recovery and purity.

As in our study, Amicosante et al. 8 evaluated different types of extraction methods in samples of SPU: phenol-chloroform, GeneReleaser kit (based on silica column) and the combination of phenol-chloroform with GeneReleaser. They concluded that the use of capture resins can significantly improve the performance of PCR tests for the diagnosis of TB. Aldous et al. 12 also achieved greater DNA recovery in SPU samples using a technique of mechanical rupture of mycobacterial membranes with glass beads. Detergent-based methods (such as the Roche assay used in the present study), however, were not able to completely remove PCR inhibitors 12,21. This may, at least in part, explain our results.

Until recently, the detection of *M. tuberculosis* was performed only by an in-house technique. This technique is time consuming and presents a high risk of sample contamination due to the manipulation of the amplicons in the post-PCR area. In our study, this technique showed good diagnostic performance when combined with the Qiagen extraction method, reinforcing the importance of the extraction technique used.

With the development of commercial kits, the Cobas Amplicor test has started to be used in routine diagnostic procedures. In this assay, the extraction process is based on the use of detergents, and the manipulation of amplicons is restricted to the post-PCR area. With the development of real-time PCR, the Cobas Amplicor test was replaced by the Cobas TaqMan test. Although this test presents no risk of contamination with amplicons, it also uses detergents in the extraction process, which can imply incomplete elimination of sample inhibitors. It is noteworthy that both assays are validated only for respiratory specimens. The Nanogen kit used in this study, which also utilizes real-time PCR, permits the detection of Mtb DNA from SPU, urine and exudates according to the manufacturer.

Although the literature regarding the performance of PCR for TB diagnosis is controversial, the results of the present study demonstrate that the three tests were able to detect low concentrations of Mtb in respiratory samples, depending on the extraction/detection methods used.

One limitation of our study is the use of samples prepared *in vitro,* which may not reflect the interaction of the mycobacteria with the biological matrix. However, similar studies have been performed by other authors, such as Klaschik et al. 22, who developed a real-time PCR protocol to screen bacteria from samples prepared *in vitro* for the accurate classification of bacteria from patients admitted to the ICU. Additionally, Opel et al. 23, in a study similar to ours, used different concentrations of PCR inhibitors to determine the efficiency in amplifying specific primers for the HUMTH01 locus. In this context, we justify the option of working with samples prepared *in vitro* as a way to gain control over the preanalytical and analytical variables assessed in the study.

In conclusion, we demonstrated that the preanalytical quality of biological samples may interfere with the diagnostic performance of molecular tests. Accordingly, the choice of the best extraction/detection method is crucial for ensuring better results, especially when we are evaluating paucibacillary samples from the respiratory tract and/or from other types of biological matrices in the clinical laboratory routine.

## ACKNOWLEDGMENTS

We thank biologist Carlos SR Silva for his assistance. Financial support: Research Support Foundation for the State of São Paulo (FAPESP) and National Research Council (CNPq), Brazil.

## AUTHOR CONTRIBUTIONS

Carnevale GG participated in the design of the study, carried out preparation of the sample sets and PCR techniques and drafted the manuscript. Vargas FS participated in the study design and coordination. Caiaffa-Filho HH helped designing the study. Acencio MM researched the conception of the study, helped in the collection of samples and in the analysis of the results. Marçal LJ participated in the collection and preparation of sample pools. Sales RK performed sample collection. Teixeira LR performed sample collection and participated in the design of the study. Antonangelo L participated in the design and coordination of the study and helped drafting the manuscript. All authors have read and approved the final version of the manuscript.

## Figures and Tables

**Table 1:** Preparation of biological samples according to the concentration of preanalytical variables evaluated.

Variables	PF	BAL	SPU
Nucleated cells (cells/mm^3^)	<3,000	<3,000	<3,000
	5,000 to 8,000	5,000 to 8,000	5,000 to 8,000
	>10,000	>10,000	>10,000
Erythrocytes (red blood cells/mm^3^)	<10,000	<10,000	<10,000
	20,000 to 40,000	20,000 to 40,000	20,000 to 40,000
	>50,000	>50,000	>50,000
Mixed	>10,000	>10,000	>10,000
	>50,000	>50,000	>50,000

PF: Pleural Fluid, BAL: Bronchoalveolar Lavage, SPU: Sputum.
